# A Systematic Review of Strength and Conditioning Protocols for Improving Neck Strength and Reducing Concussion Incidence and Impact Injury Risk in Collision Sports; Is There Evidence?

**DOI:** 10.3390/jfmk6010008

**Published:** 2021-01-12

**Authors:** Ed Daly, Alan J. Pearce, Lisa Ryan

**Affiliations:** 1 School of Science and Computing, Galway-Mayo Institute of Technology (GMIT), H91 T8NW Galway, Ireland; ed.daly@gmit.ie; 2 College of Science Health and Engineering, La Trobe University, Melbourne, VIC 3086, Australia; alan.pearce@latrobe.edu.au

**Keywords:** sport related concussion, cervical spine, neck muscle strength, injury risk

## Abstract

The objective of this systematic literature review was to evaluate the evidence regarding the development of neck strength in reducing concussion and cervical spine injuries in adult amateur and professional sport populations. PubMed, CINAHL, Science Direct, and Web of Science databases were searched systematically. The criteria for inclusion in the review were as follows: (1) a human adult (≥18 or above); (2) involved in amateur, semi-professional, or professional sports; (3) sports included involved collisions with other humans, apparatus or the environment; (4) interventions included pre- and post-neck muscle strength measures or neck stability measures; (5) outcomes included effects on increasing neck strength in participants and/or injury incidence. Database searches identified 2462 articles. Following title, abstract, and full paper screening, three papers were eligible for inclusion. All of the papers reported information from male participants, two were focused on rugby union, and one on American football. Two of the included studies found a significant improvement in isometric neck strength following intervention. None of the studies reported any impact of neck strengthening exercises on cervical spine injuries. This review has shown that there is currently a lack of evidence to support the use of neck strengthening interventions in reducing impact injury risk in adult populations who participate in sport.

## 1. Introduction

Injuries to the head and cervical spine region are common in collision sports and may result in concussion or mild traumatic brain injury (mTBI). The most recent Consensus Statement on Concussion in Sport [1] defined concussion as “a traumatic brain injury induced by biomechanical forces”. Included in these biomechanical forces are incidents such as a direct impact to the head, or an impact to another part of the body with a mechanical force transmitted to the head [2]. The Consensus Statement on Concussion in Sport [1] also states that there is an absence of clarity around whether concussion can be classified as a mTBI in a clearly defined clinical sense. While concussion may represent a subset of mTBI, it should be noted that mTBI is not a concussion and, as such, the terms should not be used interchangeably [3].

Moreover, it has been noted that, even though functional brain activation differences can persist up to two months after a concussion has been experienced, performances on standard working memory tasks are comparable to normal controls after the same length of time [4]. Adding to the complex nature of concussions, research provides support for ongoing physiological differences up to 12 months post-concussion [5]. This can manifest as ongoing fatigue that is associated with concussion symptoms as demonstrated by corticomotor and somatosensory measures using transcranial magnetic stimulation [6].

There are documented cases of the connection between collision sports, and long-term concussion injury effects in a variety of professional field sports (e.g., association football (soccer), rugby union, American Football, Australian Football, and rugby league). These associations have found a direct connection between collision impacts, concussion incidence, and long-term neurophysiological or cognitive effects associated with repeated concussion injuries [7]. For example, research in American Football has demonstrated that many former players showed high pathological evidence of chronic traumatic encephalopathy (CTE), suggesting that this condition was related to their participation in the game [8]. In association football (soccer), evidence derived from retrospective cohort studies has shown that mortality rates from neurodegenerative disease was higher compared to other common diseases among former Scottish male professional soccer players [9]. In rugby league and recently in Australian football, cases of CTE have been reported from former male professional players who had played in excess of 150 first grade National Rugby League and 400 Australian football matches during their careers. This provides persuasive evidence in support of CTE associated with repetitive head injury in professional collision sport [10,11].

The risk of concussion is highest in individuals, whether they are amateur, or elite participating in collision sports (e.g., rugby union, rugby league, soccer, Australian Rules football). Recent data highlight that head and neck injury rates in soccer account for 2.2% of all injuries in the game [12]. The rate of concussion was 0.06 concussions per 1000 h, with a 20-fold higher rate of head and neck injury during matches when compared to training sessions (rate ratio (RR), 20.2; 95% confidence interval (CI), 13.3–30.6) and a 78-fold higher rate of concussion (RR, 78.5; 95% CI, 24.4–252.5). Incidence rates of concussion in other field-based sports such as Australian Football (per 1000 player hours), ranged from 2.24 to 17.63 at elite levels of the game, and 0.35 to 14.77 at the amateur level [13].

The incidence of match concussion in elite rugby union in the UK during the 2017–18 season was 17.9 per 1000 h of match play making it the most common occurring injury in match play (RFU injury surveillance project 2017–18 [14]). The most recently available report from community rugby in the UK, reported the incidence rates during the 2017–18 as 3.6 injuries per 1000 match hours, an increase from the previous season (3.0 injuries per 1000 player match hours). The Irish Rugby Football Union (IRFU) injury surveillance report 2018/19 for amateur (non-elite) players reported injury rates for male and female players at 13% (5.7 per 1000 h of play) and 10% (4.9 per 1000 h of play) respectively, which made concussion the highest injury category in males and females during that particular season [15]. For the foreseeable future, it seems difficult to eliminate all incidences of concussion in collision sports, whether they are amateur or elite level sports [16]. This is primarily due to the existing rules, or laws that govern intermittent field sports, and the inherent physicality of the collision sports [17]. This translates into continuous exposure of serious injury risk and continued concussion risk incidence which can have a long-term impact on cognitive function to the participants involved [18]. Therefore, the focus needs to be readjusted to examine what injury mitigation strategies can be implemented to decrease the number of concussions in many collision sports.

In professional sport, neck muscle strength is postulated as a possible method to reduce cervical neck injury and concussion incidence among strength and conditioning coaches. Qualitative research by our group (currently under review) investigating methods to reduce concussion with current professional strength coaches has supported this assertion. Research in youth athletes (<18 years) has suggested that increasing isometric neck strength to stabilise the cervical spine can reduce injury risk when compared to previous seasons injury data [19]. However, the evidence to support that this type of intervention can reduce and/or mitigate against concussion in adult (elite or amateur) populations is not evident.

This systematic review therefore sought to examine the current evidence for developing neck muscle strength to reduce concussion incidence and impact injury risk in adult populations who participate in collision sport at amateur, sub elite, and elite levels.

## 2. Methods

The Preferred Reporting Items for Systematic Reviews and Meta-Analyses (PRISMA) guidelines were followed when conducting and reporting this review [20].

### 2.1. Literature Search

A literature search was carried out in the PubMed, CINAHL, Science Direct, and Web of Science databases. Terms to describe the population (e.g., adult, human) and intervention (e.g., neck muscle strengthening protocols) were included in the search strategy (see Table 1).

Criteria for inclusion in this review were: (1) a human adult (≥18 or above) population, (2) involved in amateur, semi-professional and professional sports, (3) involved in collisions with other humans, apparatus or the environment, (4) neck muscle strength intervention (5) outcomes/effects on increasing neck strength in participants and/or injury data, (6) original research, RCTs abstracts (with data/full paper not available) were included initially, and (7) written in English. Papers were excluded if they were theses (PhD, Masters or Honours), not a piece of original research; if the population consisted of animals, a paediatric age group under 18 years of age, a geriatric group who experienced mTBI due to health or illness status, if there were no outcomes of interest; or a study design of interest, or if the study was not an RCT or if it was not an intervention of interest as per the criteria. Papers were excluded where the predominant focus was on kinematics, modelling of impact, or simulated laboratory testing of impact to the head and neck.

### 2.2. Data Extraction

Data extraction was performed by two authors (ED and LR) using a tool adapted from the National Health and Medical Research Council (NHMRC) data extraction tool for RCT and cohort studies. Any disagreements were resolved by a third author (AJP). Information regarding study design, intervention and control conditions, sample size, allocation procedure, population characteristics and relevant outcomes was extracted from each paper. A meta-analysis was not feasible, hence a qualitative analysis of included studies was completed.

### 2.3. Quality Assessment

Quality assessments were performed on all included studies using a Quality Criteria Checklist for RCTs and Cohort Studies [21]. The tool consists of ten questions for which each paper is given either a ‘yes’, ‘no’, ‘unclear’ or ‘NA’ response. Research question, study population, intervention and control conditions, outcomes, study design, conclusions drawn, and funding sources were all assessed to determine the methodological quality of each paper. Papers were designated a quality rating of positive, negative or neutral based on the answers to the 10 questions. The quality rating of each paper was considered when interpreting results.

## 3. Results

**Figure 1:**
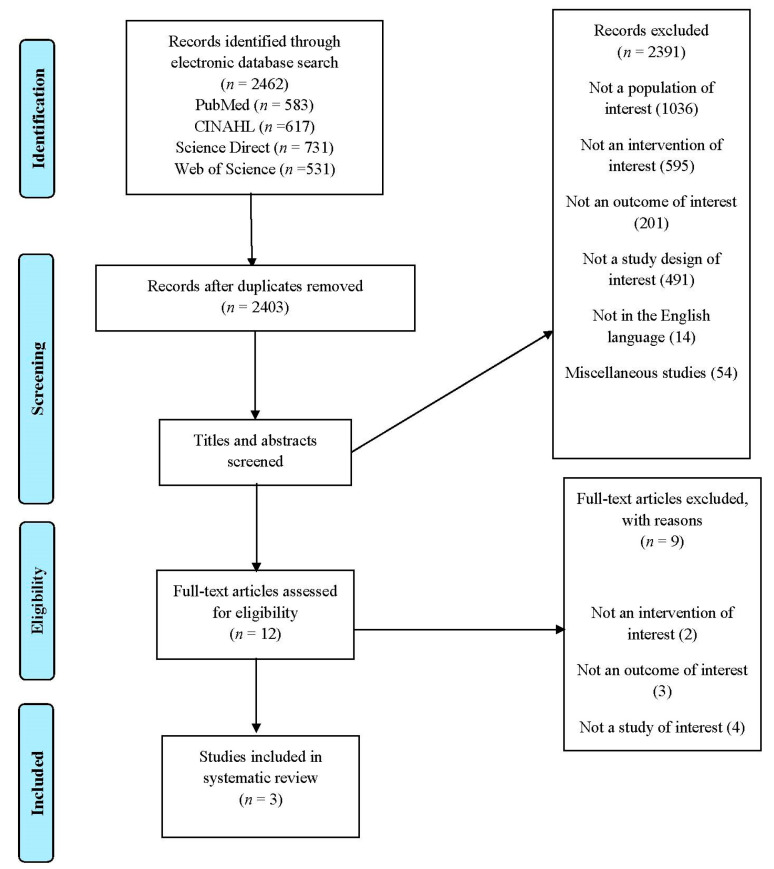
PRISMA flow diagram of included studies.

Database searches identified 2462 articles. Following title and abstract screening, 12 full papers were retrieved and assessed for inclusion. Three papers were eligible for inclusion in the review. Figure 1 illustrates the flow of studies and reasons for exclusion of full papers. All studies rated as ‘neutral’ when assessed for quality.

### 3.1. Description of Included Studies

Three studies examined the effects of a neck strengthening intervention on adult athletic populations after gathering baseline measures of isometric neck strength and re-examining neck strength after the predetermined intervention timespan (Table 2). Two of the papers [22,23] examined standard isometric neck strength as an intervention while one paper [24] examined a novel neck strengthening device. 

### 3.2. Interventions

In a study of male professional and semi-professional rugby players, Geary et al. [22] investigated outcomes after a five week intervention of specific isometric neck strength training (*n* = 15) using a commercially available digital hand held dynamometer as a measure of overall strength in all planes of motion (i.e., flexion, extension, left side flexion and right side flexion).This study included a control group (*n* = 10) who did not take part in the specific neck strengthening protocol and instead continued their normally prescribed strength program. During the five-week period, the isometric neck strengthening program was undertaken twice per week. The participants in the intervention group were required to wear a custom designed head harness and manual pressure was applied to both the head harness and participants by a professional strength and conditioning coach The participants were requested to lie supine on a standard gym bench with their feet planted on the floor and their head and neck unsupported. They were required to maintain a neutral position in their cervical spine for 10 s against the applied manual resistance of the professional strength coach. Three sets of ten second holds were performed in all planes of motion used for this intervention. The full-time professional rugby players received supervised strength and conditioning training, while the semi-professional rugby players did not receive supervised strength and conditioning support.

The study by Naish et al. [23] was 26 weeks in duration where the isometric neck strength program was focused on reducing the incidence of cervical spine injuries in the cohort (*n* = 27) selected. No control group was identified for this study, all participants were male professional rugby union players. This study used exercises that focused on isometric strength in neck flexion, neck extension, lateral neck flexion, bent over neck flexion and extension utilising a cable fly machine and a scrum machine. The neck strength intervention was initiated with a familiarisation session and followed by gathering isometric neck strength in four different direction (i.e., flexion, extension, right lateral flexion, and left lateral flexion). Peak isometric neck strength was measured using a head harness made of webbing and Velcro, the harness was attached by a cable to a load cell (HBM 2007 S40 100 kg) which was in turn attached to an immovable metal frame. Prior to the evaluation of neck strength, each participant was requested to perform three submaximal efforts (75% effort) and asked to hold these contractions for a period of five seconds. Thereafter the participants were asked to complete three, five second contractions, the highest score range of motion (ROM) of these three efforts were recorded. Rest periods (30 s) were integrated to minimise the effect of fatigue between each effort. The testing order was block randomised for this intervention.

Versteegh et al. [24] evaluated the effects of a neuromuscular neck training device on multiplanar static and dynamic neck strength. Both the intervention group (*n* = 10) and the control group (*n* = 8) were male college American football players (*n* = 10). The intervention group was trained twice per week on a novel device that used self-generated centripetal force to create dynamic rotational resistance over a seven-week period. The intervention group of players were fitted with a standard American football helmet with an added attachment on top of the helmet. On the attachment, a 25-cm rod could be extended and a weight of 125 g was located at the distal end of the rod which was parallel to the floor. The helmet was secured on the head of participants with their back unsupported and feet flat on the ground. Participants created circumduction movements of the head to initiate the weighted rod to spin on its axis while attempting to keep their bodies as still as possible. The increase in spin made the weight (125 g) provide increased resistance using centripetal force. Each participant was asked to complete six sets of fifty revolutions in a clockwise and counter-clockwise direction. The weight selection and testing protocol were established by using a sample of the of the target population. Each set was timed with a stopwatch and recorded. A portable computer used on bicycles counted the revolutions and calculated the velocity of each revolution of the set.

### 3.3. Participants

Study populations ranged from 18 to 27 participants (mean = 23). Two studies [22,23] indicated that participants were male professional or semi-professional rugby players. Geary et al. [22] included both male professional and semi-professional rugby players, where the professional rugby players (*n* = 15) acted as the intervention group and the semi-professional players (*n* = 10) were the control group for the study. The professional players were a sample of convenience and were 19.3 ± 1.3 years old, had an average height of 1.85 ± 0.06 m and were 95.2 ± 13.2 kg in mass. The control group of semi-professional rugby players were 20.7 ± 1.3 years old, had a mean height of 1.85 ± 0.03 m and an average mass of 101.3 ± 12.3 kg. No exclusion criteria were indicated in this study, all participants were members of the Ulster Rugby Academy or the Ulster Rugby A squad.

The study by Naish et al. [23] was a retrospective pre-test, post-test cohort study that involved the analysis of two years of retrospective data from a professional rugby union squad who participated in the Super 14 competition. The intervention for the 27 male players involved the integration of the neck strengthening program into an existing 26-week training program, the rationale for selecting isometric exercises was to reduce risk of injury to cervical discs, neural structures and facet joints. The cohort of players had an average age of 25.2 ± 3.9 years, the average height was 187.1 ± 0.06 m and had a mean mass of 102 ± 11.9 kg. The cohort in this study had a split of 15 forwards and 12 backs that were consistent over the two seasons that the data was gathered.

Versteegh et al. [24] used a cohort of 18 male college students who played American Football, the purpose of the intervention was intended to target the ability of the neck muscles to perform coordinated multiplanar plyometric contractions. The intervention group was selected to reflect the anthropometry of the intervention group where possible. The control group was aged 20.8 ± 1.8 years, compared to the control group whose average age was 20.8 ± 1.4 years. Body mass was similar between both groups where the intervention group had a mean mass of 113.9 ± 20.2 kg and the control group had an average mass of 112.4 ± 21.2 kg. In terms of height, the intervention group was 1.90 ± 0.056 m and the control group had an average height of 1.88 ± 0.062 m.

### 3.4. Outcome Variables

The primary aim of the study by Geary et al. [22] was to investigate how effective a specific neck strengthening intervention would be on the isometric neck strength profile of professional male rugby players. They found that there were no significant between-group differences in isometric neck strength musculature noted preintervention [22]. After the specific neck strengthening intervention, it was noticed that there was a significant main effect for time observed (*p* < 0.05). This was recorded in the intervention group as an increase in isometric neck strength in all planes after the five-week intervention (flexion preintervention = 334.45 ± 39.31 N vs. flexion postintervention 396.05 ± 75.55 N; extension preintervention = 606.19 ± 97.34 vs. extension postintervention = 733.88 ± 127.16 N; left side flexion preintervention = 555.56 ± 88.34 N vs. left side flexion postintervention = 657.14 ± 122.99 N; right side flexion preintervention = 570.00 ± 106.53 N vs. right side flexion postintervention = 668.006142.18 N) [25]. It was noted that there was no significant improvement in neck strength observed for control group participants. This study stated that it was plausible that a neck strengthening intervention may be effective in reducing neck injuries particularly in physical contact areas during training and game time [22].

The primary outcome variables in the study by Naish et al. [23] included the number and type of cervical spine injuries as well as the severity of these types of injuries. The severity was measured by the number of days that the players were considered unavailable for matches and training. Cervical spine injuries that did not result in time loss for matches and training were not examined as part of this study. Characterization of the severity and overall diagnosis of cervical spine injuries were carried out by two senior physiotherapists by means of a clinical examination. Once a cervical spine injury was recorded, the details were entered into a database using the Orchard Sports Injury Classification System (OSICS), using this system, injuries were recorded using four specific codes; (1) cervical spine facet joint injury (NJXX), (2) cervical disc prolapse (NCLP), (3) cervical facet joint pain/chronic inflammation (NJPX) and (4)cervical disc sprain (NCLX).

Secondary outcome variables for the study [23] included the baseline testing of isometric neck strength at the start of the season and at week 5 of the intervention. Retesting at week 5 was completed by the professional strength & conditioning staff to observe any acute increases in neck strength during the initial five-week period. The rationale for continuing the neck muscle strength training was based on favourable data from the first 5 weeks which then continued for the remainder of the season of 26 weeks duration.

Following the intervention in this study [23], there were no significant differences evident between seasons for the number of players with cervical spine injury (eight players in 2007–2008, 6 players in 2008–2009, *p* = 0.75) or the total number of cervical spine injuries (12 and 6 for the 2007–2008 and 2008–2009 seasons respectively, *p* = 0.34). Two players had one or more recurrent disc injuries. While there was no significant difference (*p* = 0.18) evident between-years for the number of training injuries (one in 2007–2008 and four in 2008–2009) there was a significant reduction (*p* = 0.03) in the number of cervical spine injuries experienced in matches (from 11 in 2007–2008 to two in 2008–2009). The time loss related to these injuries was not significantly different (*p* = 0.40) between-season. Specifically, there was no significant difference (*p* = 0.20) in the days lost from training in 2007–2008 (21 days) and 2008–2009 (17 days) and there was no significant difference (*p* = 0.14) for the number of days lost from matches in 2007–2008 (79 days) and 2008–2009 (23 days). This study recorded non-significant increases in isometric neck strength in all ranges of motion tested, with no significant increases in isometric neck strength recorded.

Versteegh et al. [24] examined the primary outcome of the training effect of a novel neuromuscular strengthening protocol on dynamic and static neck strength in a group of male college American football players. From this study [24], it was concluded that there was a composite neck strength improvement in the intervention group when compared to the control group. The mean change in composite strength of the intervention group was 32 N (95% CI, 13–50), whereas in the control group, it was 12 N (95% CI, 210 to 34). Change in axial rotation strength the direction of most interest, demonstrated the largest mean difference between the control and intervention cohorts of 46 N (95% CI, 9–83) and the largest effect size with 95% CI. There was retention inconsistency between groups, when a sensitivity analysis was applied, in which only eight control subjects were selected as matched to the intervention group, it revealed nearly identical findings to the full sample. All performance parameters showed a qualitative improvement over the course of the seven weeks of training protocol.

It is interesting to note that both the intervention group, and the control group continued with a traditional neck strengthening on a straight-plane, isotonic, four-way neck machine in conjunction with the novel multiplanar device for the intervention group. After seven weeks intervention period, the researchers recorded a large positive effect size (Hedge’s g, 0.68) in isometric composite neck strength relating to the intervention cohort when compared to the control group (difference, 20 N; 95% CI, 28 to 48). This study [24] concluded that dynamic training for neck strength may reduce injury risk in concussion or other injury to the head–neck segment.

## 4. Discussion

This systematic review of the literature attempted to evaluate evidence regarding neck strengthening protocols as a modifiable factor in reducing cervical neck injuries and concussion in adult sports populations. This review has highlighted the dearth of evidence in adult populations for prospective interventions designed to reduce concussion incidence and cervical spine injuries. In recent years, it has been proposed that an increase in cervical neck strength may act as a mitigating factor in reducing concussion incidence [26]. It has further been postulated that impact location and magnitude could have a greater influence on rotational movement of the head than cervical muscle state [27]. Other evidence has found that the connection between stabilising the cervical spine area of players and reducing injury risk remains unsubstantiated and requires further research [19]. Because of an inconclusive position in adult populations (i.e., whether neck strength does mitigate against cervical spine injury or concussion), it remains important to evaluate novel methods for injury reduction.

Physical impact with opposing players or with the surrounding playing environment plays a part in head acceleration. Therefore, any novel method that influences head acceleration could conceivably mitigate against the physical mechanisms of concussion and cervical spine injury. Recently, it has been discussed that developing neck muscle strength could influence the multi directional movement of the head in contact sports by increasing neck muscle stiffness in an attempt to ‘brace for impact’ [28].

A considerable body of research is required to offer a comprehensive answer to this multifactorial issue. Improving the levels of neck strength as a method to reduce injury risk in adult populations as discussed in the three papers included in this review must be viewed with caution. There is evidently a lack of power in the sample sizes as suitably powered sample sizes are difficult to attain for elite athlete populations.

### 4.1. Interventions to Improve Neck Strength

From the results of the research by Geary et al. [22], it is not possible to interpret whether the intervention brought about a reduction in the numbers of cervical spine injuries in the participants. This study did not address the positional or unit specific requirements for players of rugby union. There are significant differences from a physiological point of view between rugby union forwards and rugby union backs [29]. This study examined, sagittal plane and frontal plane isometric neck strength alone, it is acknowledged that these two planes of motion are not the only contributing factors contributing to overall neck stability. In conjunction with this, the study [22] did not evaluate how the strength program affects proprioception or motor control in the segmental cervical spine. This study [22] did demonstrate that there can be improvements in neck strength over a relatively short period of time. However, it did not demonstrate that the improvements in neck strength would have an effect on reducing cervical neck injury.

### 4.2. Retrospective Analysis

Naish et al. [23] demonstrated that after a neck strength intervention, there was no significant difference (*p* = 0.18) evident between playing seasons for the number of injuries occurring in training, or no significant difference (*p* = 0.20) in the days lost from training for this cohort of professional players. These findings indicate that the introduction and implementation of neck strength development techniques across adult playing populations warrants further comprehensive investigation. Limitations evident from this study were that it was a retrospective analysis rather than a prospective study design such as a randomised control trial. Further limitations associated with the study [23] was the reduced number of common participants across the seasons that were analysed. It must be acknowledged that this study dealt exclusively with elite level athletes and for this reason, the findings may not be applicable to rugby union at amateur or community level players. Furthermore, this study [23] focused their protocol solely on isometric contractions using flexion, extension and lateral flexion to the exclusion of contractions utilising other methods such as dynamic contractions, plyometric contractions or impact anticipation.

### 4.3. Highly Trained vs. Amateur Athletes

Versteegh at al. [24] had similar limitations whereby the participants were a group of highly trained male athletes and evidence from this study may not be applicable to the general population or non-elite athletes. From this study, it was reported that measurement bias may have occurred because the participants were not blinded to the neck muscle strengthening training program. This may have led to the intervention group applying increased effort into their post training assessments. This study was also limited by sample size and had a limitation in terms of the manipulation of the duration for the training program for the participants involved. Further limitations were evident in relation to the manipulation of sets and repetitions which may have produced a more noticeable effect on the dose–response relationship in this intervention.

### 4.4. Protocols to Improve Neck Strength

The interventions from the three studies included in this review have shown that strength and conditioning protocols can improve neck muscle strength. However, the findings reported are from a narrow focus of sports activity, namely rugby union and American football. Also, the included studies focused on male populations only. This is a concern as emerging evidence is reporting that female athletes are at greater risk of concussions, reporting greater symptom severity and are reporting longer recovery times [30]. Investigation into the possibility of neck strength interventions as a modifiable factor to mitigate against cervical spine injury or concussion in female sport participants is required.

### 4.5. Follow Up Research

A noticeable limitation of the available evidence is whether neck strength interventions manage to protect the players once they have engaged in these types of interventions. There is an absence of follow up research to demonstrate the conclusive effectiveness of these types of interventions in the reduction of cervical spine injury and concussion. Additional research has attempted to review neck strength from alternative perspectives under controlled conditions. For example, one study [31] sought to examine the effect of the kinematic response of the head in controlled laboratory conditions. Kinematic studies are useful to determine responses but cannot provide support for a recommendation that strength training of the neck musculature is an effective strategy to mitigate against injury in contact sports.

### 4.6. Limitation of the Search Strategy

The search strategy for this review was aimed specifically at adult populations (male and female) involved in sports activity and excluded studies associated with paediatric populations (participants <18 years old) and geriatric populations. There is evidence to suggest that neck strength measures can be utilized as a screening tool in adolescent populations based on neck circumference and mean head to neck circumference ratio. Collins et al. (2014) [32] proposed that an increase in neck strength can be used as a means to reduce the odds of concussion risk in high school populations who are engaged in specific contact sports. Their study employed univariate logistic regression to assess unadjusted odds of concussion for derived anthropometric measurements, age, gender, body mass index (BMI), and sport. Their results found that neck strength (*p* < 0.001), gender (*p* < 0.001), and sport (*p* = 0.007) were significant predictors of concussions in unadjusted models. This study adds weight to the proof of concept that neck strengthening may improve concussion risk but again, similar to the studies in the adult population, has not employed an intervention to test this hypothesis. An alternative study has examined isometric neck strength interventions in youth populations which have focused on isometric neck strength with the exclusion of dynamic neck strength or collision anticipation [33]. It is unclear if studies of this kind in youth populations could translate to adult populations based on age, anthropometric and gender differences. A subsequent limitation of this review was the exclusion of studies where there was a combination of populations (i.e., cohorts that included populations <18 years of age and >18 years of age in the same study).

### 4.7. Summary

The evidence in this systematic literature review supports the most recent Consensus Statement on Concussion in Sport [1] which states that prevention interventions using modifiable risk factors remain unclear in terms of possible interventions to prevent or reduce the risk of concussion in sport. Whether we examine neck strength interventions using gender, age, or under specific category of sports parameters, there is evidence to suggest that athlete education programs can be utilised as a strategy to reduce concussion risk in sport [25,34,35]. Reporting rates from playing populations and coaches have been improving due to an increase in knowledge and information about concussion [36,37,38,39]. However, many incidences of concussion are subjectively reported, consequently there are still discrepancies in understanding the effects of concussion due to misinterpretation of subjective self-reporting, particularly in amateur sports [40,41]. The research in relation to the effects of concussion in female sports is under researched in relation to the epidemiology of female concussion rates, symptoms and cognitive recovery post-injury [42]. Gaining a more comprehensive understanding of the aetiology of female concussion must be seen as a priority for female sports participants, in relation to symptoms, and inflammatory biomarkers after experiencing concussion [43].

## 5. Practical Applications

Strength and conditioning coaches often cite neck strengthening exercises as a means to mitigate against concussion risk and injury to the cervical spine. This systematic review has shown that research on neck strengthening exercises in adult populations reducing cervical neck injuries and concussion is currently limited. A substantial body of research focuses on head acceleration, rotational forces of the head, and impact measurement to the head. However, these interventions do not specifically offer adequate evidence towards reducing concussion incidence in adult populations. The evidence suggests the need for more research to be conducted in both males and females, in larger samples sizes, with longer follow up periods to test the efficacy of neck strengthening as a means of mitigating the effects of concussion and cervical spine injuries in impact or collision sports.

## Figures and Tables

**Table 1:** Sample search strategy terminology.

Search Concept/Term	Synonyms
Neck strength	cervical neck muscle strength OR sternocleidomastoid muscle strength OR musculus sternocleidomastoideus OR cervical range of motion OR isometric neck muscle strength OR neck flexor musculature OR neck muscle strength endurance OR neck muscle strength testing OR active neck muscle training OR neck muscle size OR neck strength measurements OR neck muscle coactivation
Concussion and/or mTBI	concussion OR head injury OR head trauma OR sub concussive injury OR head impact OR brain injury OR head trauma OR neuroimaging biomarkers OR neuropsychological testing OR eye movement OR cognitive function

**Table 2:** Study design, population, intervention and control conditions, duration and outcomes measures of studies.

Study (Author, Year, Location)	Quality Rating	Intervention Group	Intervention	Control Group	Duration	Outcome	Results
Geary et al., 2014. Ireland [22]	N	15 male professional rugby union players. (mean ± SD age = 19.33 ± 1.29 years; height 1.85 ± 0.06 m; body mass = 95.15 ± 13.24 kg)	Participants were required to lie supine on a standard gym bench with their feet planted on the floor and their head and neck unsupported. A manual pressure was applied in each direction (flexion, extension, left-side flexion, and right-side flexion) by a professional strength and conditioning coach with the participant being required to maintain their cervical spine in a neutral position for 10 s against the applied manual resistance. In total, three 10-s holds were performed in each direction.	10 semi-professional male rugby union players (mean ± SD age = 20.70 ± 1.25 years; height = 1.85 ± 2.74 m; body mass = 101.30 ± 12.32 kg)	5-week neck strengthening program performed twice per week.	Isometric Neck Strength	No significant between-group differences in isometric neck strength were noted preintervention. A significant main effect for time was observed (*p*, 0.05) -the intervention group increased isometric neck strength in all planes after the 5-week intervention (F preintervention = 334.45639.31 N vs F postintervention396.05675.55 N; E preintervention = 606.19697.34 vs E post-intervention = 733.886127.16 N; LSF preintervention = 555.56688.34 N vs LSF postintervention = 657.146122.99 N; RSF pre-intervention = 570.006106.53 N vs RSF postintervention =668.006142.18 N). No significant improvement in neck strength was observed for control group participants.
Naish et al. 2013. Australia [23]	N	27 male players consisting of 15 forwards and 12 backs (mean ± SD age = 25.2 ± 3.9 years, height 187.1 ± 6.3 cm and mass, 102 ± 11.9 kg).	A progressive and supervised isometric neck strengthening intervention program was added to the overall strength and conditioning program at the beginning of the 2008–2009 pre-season period. Isometric neck strengthening exercises were selected as it was believed that the absence of movement was likely to be of less risk to the cervical disc, facet and neural structures. Exercises that involved producing an isometric contraction directed in axial rotation were not included	No control group was identified	26-week program two phases; (1) a 13-week strengthening phase followed by (2) a 13-week maintenance phase	Isometric neck strength Reduction in cervical spine injuries	No significant differences evident between seasons for the number of players with cervical spine injury (8 players in 2007–2008, 6 players in 2008–2009, *p* = 0.75) or the total number of cervical spine injuries (12 and 6 for the 2007–2008 and 2008–2009 seasons respectively, *p* = 0.34). The number of cervical spine injuries experienced in matches decreased (from 11 in 2007–2008 to 2 in 2008–2009). The time loss related to these injuries was not significantly different (*p* = 0.40) between-season. The initial 5-week neck strengthening program resulted in a non-significant increase in isometric neck strength in all four directions of movement (flexion, *p* = 0.271; extension, *p* = 0.481; left lateral flexion, *p* = 0.687; right lateral flexion, *p* = 0.711)
Versteegh et al. 2019. Canada [24]	N	8 male players mean ± SD − neck girth (cm) 43.8 ± 2.3 Age (y) 20.8 ± 1.4 Height (m) 1.88 ± 0.062 Body mass (kg) 112.4 ± 21.5	Quasi experimental pilot study design with intervention (*n* = 8) and control (*n* = 10) groups. The intervention group was trained (twice/week,10 min, for 7 weeks) on a training device that uses self-generated centripetal force to create a dynamic rotational resistance. The protocol was intended to target the ability of the neck muscles to perform coordinated multiplanar plyometric contractions. Both groups also continued with traditional neck strengthening that included training on a straight-plane, isotonic, 4-way neck machine.	10 male players mean ± SD − neck girth (cm) 43.5 ± 3.0 Age (y) 20.8 ± 1.8 Height (m) 1.903 ± 0.056 Body mass (kg) 113.9 ± 20.2	7 weeks	Dynamic and static neck strength	Composite neck strength improvement favoured the intervention group. Mean change in composite strength of the intervention group was 32 N (95% CI, 13–50), whereas in the control group, it was 12 N (95% CI, 210 to 34). Performance on the training device showed improvement after routine practice within 1 week, as evidenced by a trend toward increased peak speed in revolutions per minute (RPM). After 7 weeks for the intervention group, peak RPM increased from 122.8 (95% confidence interval [CI], 91.3–154.4) to 252.3 (95% CI, 241.5–263.1).
